# Dental Hygiene

**Published:** 1890-04

**Authors:** 


### Editorial.

### DENTAL HYGIENE.

The past decade has marked an era of advance in our knowledge regarding the etiology of dental diseases, until now the terms sepsis, antisepsis, infection, and disinfection are as common in our literature as pulp conservation and mechanical dentistry formerly were, and yet how many of us use the terms intelligently? How many can explain the pathology of alveolar dental abscess, pyorrhœa alveolaris, etc., according to the new school of dentistry?

The rapid advance made in our knowledge of the etiology of dental diseases makes it necessary to revise the teachings which have largely held up to the present day.

When it is understood that nearly if not all the diseases pertaining to the oral cavity are the result of unhygienic conditions, then the importance of the subject will be better appreciated. The mouth is known to be a hot-bed in which nearly all forms of micro-organisms find a suitable habitat and culture media. It is true that all are not pathogenic, that is, disease-generating, but many such do find their way into the oral cavity, and from thence into the alimentary canal, the lungs, and through the mucous membrane into the tissues of the body, thereby producing local or general infection. Nearly all the diseases of the mouth sooner or later become complicated by the presence, if not the direct action, of germ-life. Not a few owe their origin to micro-organisms. Suppuration of the gums and the pulp, as also alveolar abscess, is due in most cases to direct infection from the oral cavity. That stage of Riggs's disease which is known as pyorrhœa alveolaris is undoubtedly caused by infection. The disease may, and probably does, begin as a catarrhal process, but the stage where pus is formed indicates the presence of some one of the pus-forming micro-organisms: as it is now generally admitted that pus is formed through the action of certain well-known germs. Then decay of the teeth themselves, as has been so ably demonstrated by Dr. Miller, is caused by acid fermentation in contact with unexposed surfaces of the teeth.

The mouth forms the open portal to the system. The teeth stand as sentinels to guard the entrance, and their care should form the most important subject for discussion in our literature. It is true that we do devote most of our time to their conservation after they have been to a greater or less extent destroyed by the influence of unhygienic conditions, but knowing as we now do the etiology of so many of the diseases pertaining to the mouth and teeth, it becomes our bounden duty to give more attention to informing ourselves and patients regarding the prophylactic measures necessary to prevent their inception. The loss of a tooth, in the light of our present knowledge, should be looked upon as an evidence of incapacity upon the part of the operator, provided he has the full confidence and co-operation of the patient. Children should be sent to the dentist from early childhood and all irregularities corrected and all pits and fissures filled to prevent decay and full directions given as to the care of the teeth. If the patient implicitly follows these we can promise him that he will be able to retain his teeth throughout his natural life with only now and then a filling, a desideratum not to be despised.

In view of the fact that the subject of oral hygiene has not been as fully treated in our dental literature as its importance demands, it seems that there is room for some elementary work in this direction, so that the busy practitioner may acquire the needed instruction which will enable him to understand the phenomena with which he daily comes in contact, so as to intelligently explain the same to his patients without having to spend his leisure hours in painstaking experiments.

